# Accurate *ab initio* prediction of NMR chemical shifts of nucleic acids and nucleic acids/protein complexes

**DOI:** 10.1093/nar/gku1006

**Published:** 2014-11-17

**Authors:** Andrea Victora, Heiko M. Möller, Thomas E. Exner

**Affiliations:** 1 Department of Chemistry and Zukunftskolleg, Universität Konstanz, 78457 Konstanz, Germany; 2 Institute of Chemistry, University of Potsdam, Karl-Liebknecht-Strasse 24-25, 14476 Potsdam OT Golm, Germany; 3 Institute of Pharmacy, Eberhard Karls Universität Tübingen, Auf der Morgenstelle 8, 72076 Tübingen, Germany

## Abstract

NMR chemical shift predictions based on empirical methods are nowadays indispensable tools during resonance assignment and 3D structure calculation of proteins. However, owing to the very limited statistical data basis, such methods are still in their infancy in the field of nucleic acids, especially when non-canonical structures and nucleic acid complexes are considered. Here, we present an *ab initio* approach for predicting proton chemical shifts of arbitrary nucleic acid structures based on state-of-the-art fragment-based quantum chemical calculations. We tested our prediction method on a diverse set of nucleic acid structures including double-stranded DNA, hairpins, DNA/protein complexes and chemically-modified DNA. Overall, our quantum chemical calculations yield highly/very accurate predictions with mean absolute deviations of 0.3–0.6 ppm and correlation coefficients (*r*^2^) usually above 0.9. This will allow for identifying misassignments and validating 3D structures. Furthermore, our calculations reveal that chemical shifts of protons involved in hydrogen bonding are predicted significantly less accurately. This is in part caused by insufficient inclusion of solvation effects. However, it also points toward shortcomings of current force fields used for structure determination of nucleic acids. Our quantum chemical calculations could therefore provide input for force field optimization.

## INTRODUCTION

Nuclear magnetic resonance (NMR) chemical shifts are highly sensitive probes for the structural features around the corresponding nuclei and in this way for the conformations of biological macromolecules like proteins and nucleic acids. Predictions are, therefore, very valuable for a variety of applications including structure validation, automatic assignment and even structure prediction. This has been recognized for proteins some time ago and many approaches exist for calculating chemical shifts using empirical (1–7) or *ab initio* methods (8–33). For nucleic acids, the use of NMR chemical shifts could be even more advantageous. On the one hand, NMR structure determination of these molecules suffers from the scarcity of accessible nuclear Overhauser effect (NOE) derived distance constraints and the complicated interpretation of these few constraints caused by the high degree of flexibility especially in RNA (34). On the other hand, quantum chemical calculations on small test systems like mono-nucleotides, tri-nucleotides or base pairs showed that sugar ring puckering (35–39), exocyclic and glycosidic torsions (37,38,40), hydrogen bonds between base pairs (41–43) and ring current effects brought about by base stacking (44–47) have a profound influence on the chemical shifts. These can be used to predict structural features (46–51). One early approach of Altona *et al*. (48) approximates the chemical shift within B-helices by additive parameters just depending of the type of the central as well as nearest-neighbor residues. The SHIFTS program by Case *et al*. (51), NUCHEMICS by the group of Wijmenga (46) and the approach of Sahakyan and Vendruscolo (47) use empirical models for the ring current and electrostatic effects. Frank *et al*. (50) compliment the properties from NUCHEMICS by additional structural descriptors like torsions and hydrogen bonds in a machine learning approach based on random forest. The NUCHEMICS approach (46) is also used e.g. together with the method of Altona *et al*. (48) for a web server predicting chemical shifts of B-helices (49) and in NMR-guided structure optimization/determination based on molecular dynamics (34) or the Rosetta approach (CS-Rosetta-RNA) (52).

These empirical or semi-empirical methods clearly have the advantage of speed. However, there are also clear disadvantages. The very limited statistical data basis precludes an application of empirical and semi-empirical methods to non-canonical DNA/RNA structures, complexes with proteins or other types of ligands or chemically-modified structures. Furthermore, even for canonical double stranded nucleic acids assigned chemical shifts of non-proton-nuclei are still rare limiting the reliability of empirical methods targeting these nuclei.

Due to their *ab initio* foundation, quantum chemical calculations based on the QM/MM methodology or fragmentation approaches do not have these disadvantages and they can be applied to all atoms of canonical and non-canonical structures. Whereas systematic investigations encompassing larger test sets have been reported for peptides and proteins (13,20), to our knowledge, comparable studies are lacking in the field of nucleic acids.

*Ab initio* calculations on single non-canonical structures can be found in the literature, e.g. on a DNA triplex by Barfield *et al*. (53) and on interstrand cross-linked DNA by Pauwels *et al*. (54). A more detailed analysis with respect to structural influences on phosphorus chemical shifts was performed by Sklenar using density functional theory (DFT) combined with molecular dynamics calculations (55–58). Here, we report on the calculation of proton chemical shift for a diverse set of systems based on our fragmentation approach. This includes canonical DNA duplexes with and without modified residues, a DNA/RNA hybrid, a hairpin structure, a riboswitch as well as two DNA–protein complexes. The overall excellent results combined with the possibility to explain deviations between calculated and experimental values by structural or dynamic features renders the method highly valuable for structure validation. Additionally, these calculations constitute the benchmark for the further improvement of the method by e.g. including solvent effects and conformational averaging.

## MATERIALS AND METHODS

**Figure 1.**
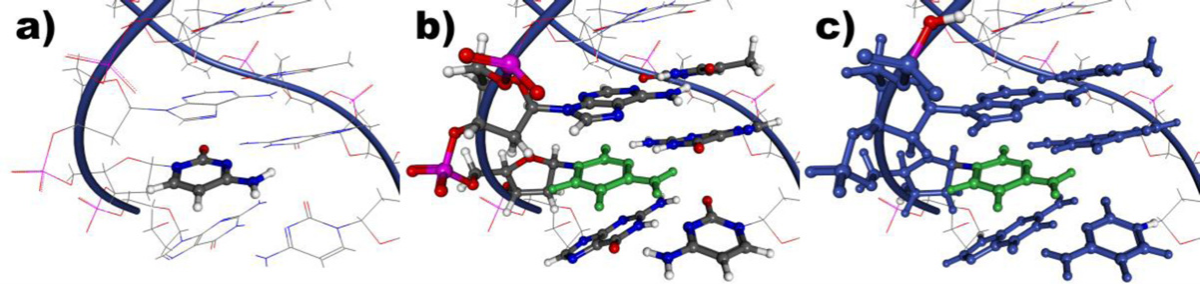
Visualization of the fragmentation approach: (**a**) The molecule is divided into independent fragments. (**b**) Each fragment, a cytosine base in this case, is then extended by surrounding atoms up to a specific distance. (**c**) Finally, broken bonds are saturated by capping groups.

To predict NMR chemical shifts of larger biomolecules such as the nucleic acids described here with a reasonable computational demand using *ab initio* methods, the system has to be divided into subsystems, which can then be calculated independently. One such fragmentation approach based on the field-adapted adjustable density matrix assembler (FA-ADMA) method has proven very successful for calculating chemical shifts in proteins (20,59,60). Only minor modifications were needed to treat nucleic acids. The main idea is to subdivide the target molecule into a set of mutually exclusive families of nuclei defining the molecular fragments. For each of these, a parent molecule is constructed by surrounding the fragments by additional regions, called surroundings in the following discussion, with the same local nuclear arrangement as in the macromolecule. This subdivision is shown for a specific example in Figure 1. A part of the backbone including one phosphate and one sugar moiety or a nucleobase constitutes a fragment. All neighboring fragments, for which at least one atom is closer to one atom in the central fragment than a defined distance, is added completely to the surroundings. For all calculations presented in this study, a distance of 5 Å was chosen. To saturate broken bonds resulting from the fragmentation at the border of the surrounding, capping groups are added in form of a single hydrogen atom or an OH group. The fragment, the surroundings and the capping groups, combined are called a parent molecule. These parent molecules are then used as explicit quantum chemical part in the fragment calculations. The remaining parts are added in the calculations as partial charges. The proteins in complex with the nucleic acids are segmented as described before (20,59) with the backbone or the side-chain atoms of one amino acid building one fragment. The ligand neomycine was divided into its different ring systems and aliphatic substituents. To limit the number of fragments, substituents with only one heavy atom were added to the corresponding ring.

The independent calculations for each parent molecule were performed with the Gaussian09 program package (61) and the GIAO (gauge invariant/including atomic orbitals) formalism (62–65). DFT with the mPW1PW91 functional (66) and the 6–311g(d) basis set (67,68) were employed. This combination has been shown to provide the best computational demand/performance ratio in protein calculations. The partial charges representing the additional part of the molecule were calculated with the Gasteiger-Hückel method (69). Polarization effects of the surrounding water were approximated with an implicit solvent model (IEF-PCM (70–72)) based on the self-consistent reaction field (SCRF). The cavity for the solute inside the high dielectric constant describing the solvent is at the moment only defined by the quantum mechanical atoms (fragment and explicit surroundings). This description of having the partial charges embedded in the high dielectric medium, even if not completely satisfactory, led to significant improvements in the protein calculations and was, therefore, also adopted here. An alternative approach in which the cavity is being increased by using pseudo atoms is currently in development. From these calculations, the isotropic chemical shieldings of the inner fragment were extracted and compared to the experimental chemical shifts ($\delta _{\exp }$). For the visualization of the errors in the calculations, the isotropic chemical shieldings ($\sigma _{{\rm calc}}$) had to be converted to chemical shifts ($\delta _{{\rm calc}}$) by $\delta _{{\rm calc}} = \sigma _{{\rm Standard}} - \sigma _{{\rm calc}}$ using the isotropic shielding ($\sigma _{{\rm Standard}}$) of the corresponding standards, tetramethyl silane (TMS) in the case of protons. $\sigma _{{\rm Standard}}$ was determined as the averaged sum of the experimental chemical shift and the calculated chemical shielding (internal standard, $\sigma _{{\rm Standard}} = \frac{{\sum {\delta _{\exp } - \sigma _{{\rm calc}} } }}{n}$ with *n* as the number of experimentally determined proton shifts). An alternative approach, mostly used for small systems, is to calculate the isotropic chemical shift of the standard directly using the same level of theory (external standard). However, the advantage of the method used here is that it removes systematic errors in the calculations inherent to the chosen level of theory/basis set combination or caused by an inconsistent treatment of the standard. Such effects are more relevant for ^15^N chemical shifts and when including explicit solvents and conformational averaging, respectively (20). Consistently, the deviations in ^1^H chemical shifts calculated by the different methods (internal vs. external standard) is only in the range of 0.1 ppm for the systems discussed here. Additionally, small offsets sometimes seen in experimental spectra are removed using the internal standard.

## RESULTS AND DISCUSSION

As described in the introduction, the goal of this study was to evaluate the state of the art of *ab initio* NMR chemical shift prediction of nucleic acids using a large set of test examples including canonical and non-canonical structures. These range from small, well-defined systems like short duplexes and a hairpin over structures including modified nucleotides or small ligands-RNA to protein–DNA complexes. Additionally, two G-quadruplex conformations were chosen as representatives of Hoogsteen base pairs, which cannot be predicted using empirical methods at the moment. Information on the specific systems as well as the mean absolute deviation (MAD) in the calculated chemical shifts for the fragment calculations are summarized in Table 1.

**Table 1.** Structural information, MAD between experiment and prediction, and computational demand (given for 1 core of a 2 Quad-Core Intel Xeons with 2.8 GHz node) for the 10 systems investigated in this study

Name	PDB entry	Models used from PDB entry	Number of experimental chemical shifts	MAD/ppm	CPU core hours per model
DNA duplex including the potent anti-poxvirus agent cidoflovir	2L8P	1	162	0.496	1300
d(GCGAAGC) hairpin	1KR8	1–10	68	0.501–0.540	500
Two lac repressor molecules bound to their natural operator O1	1L1M DNA	1	358	0.670	26 150
(3 + 1) G-quadruplex formed by hTERT promoter sequence	2KZD	1–10	163	0.653–0.808	2700
all-parallel-stranded G-quadruplex formed by hTERT promoter sequence	2KZE	1–10	150	0.560–0.805	2300
d(CGTACG)_2_	1K2K	1	45	0.387	750
d(TGATCA)_2_	1SY8	1–10	47	0.339–0.378	800
borano-phophate modified DNA/RNA hybrid	2LAR	1	130	0.489	1520
riboswitch N1 with bound ribostamycin	2KXM	1	248	0.541	5580
antennapedia homeodomain–DNA complex	1ADH DNA	1	258	0.421	10 350

For the protein–DNA complexes 1L1M and 1ADH, MADs are provided for the DNA part,only, whereas the computational demand listed pertains to complete complex.

For the larger systems, only the first model was used for the prediction to limit the computational demand (last column of Table 1) The main findings will be illustrated with five challenging systems in the following. A more detailed description of the additional examples can be found in the supporting information.

### Double helices: DNA duplex including the potent anti-poxvirus agent cidofovir

**Figure 2.**
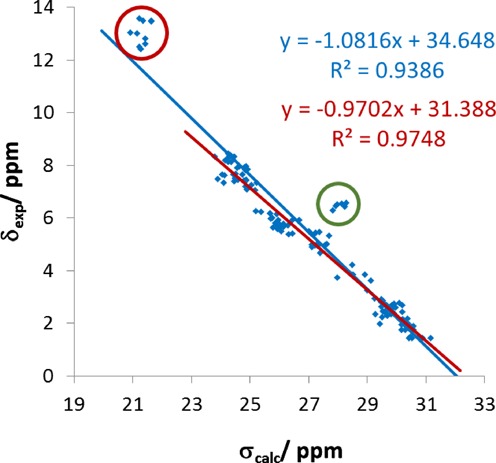
Correlations of the calculated isotropic chemical shielding with the experimental chemical shifts for a DNA duplex containing the potent anti-poxvirus agent cidofovir (PDB entry 2L8P, BMRB entry 17422): Correlation lines based on all nuclei (blue line) and neglecting the two problematic groups (red line, neglected nuclei in red and green circles) are shown.

**Figure 3.**
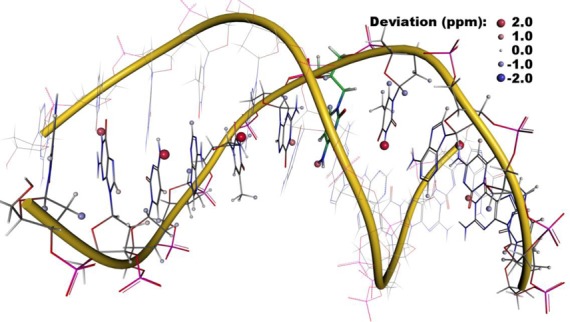
Spatial distribution of errors in the calculated ^1^H NMR chemical shifts for the first chain of a DNA duplex containing the potent anti-poxvirus agent cidoflovir (PDB entry 2L8P): Cidoflovir is marked in the middle with green carbon atoms. The nuclei of the two groups, which are predicted high-field-shifted compared to the experiment, are clearly seen as the large red spheres with errors larger than 1–2 ppm.

We chose the DNA duplex containing the potent anti-poxvirus agent cidofovir (PDB entry 2L8P, BMRB entry 17422) as studied in the group of Sykes (73) as our first example. Cidofovir is an acyclic nucleoside phosphonate and is incorporated by viral polymerases replacing dCTP. The studied construct is composed out of 12 bp with nucleotide 7 of the first chain replaced by cidofovir. To restrict the computational demand, we concentrated only on the first of the 10 models stored in the PDB entry. The proton chemical shift predictions are of excellent quality with a squared correlation coefficient *R*^2^ = 0.9386 and a slope of −1.0816 (see Figure 2), where the latter is very close to the optimal value of −1. The visualization of the errors mapped onto the molecular structure (the first and the second chain is shown in Figure 3 and the supporting information, respectively) highlight a number of nuclei, for which the chemical shifts are predicted at a too high field by more than 1–2 ppm. The first group of nuclei, marked by a green cycle in Figure 2, are protons of the amino group of cytosine forming hydrogen bonds to the solvents. The insufficiency of the implicit solvent model to predict the influences of hydrogen-bonded water molecules was already described for proteins (60), where polar protons were also predicted to resonate at too high field. The second group marked by the red cycle are protons of guanine or thymidine forming the central hydrogen bonds in the base pairs. These appear in the experimental spectrum between 12 and 14 ppm while the calculated maximum is at 11.5 ppm. Such extreme high values were only calculated for very short hydrogen bonds of <1.6 Å between N-methyl acetamide with surrounding explicit water molecules (60,74). Therefore, we speculate that the optimization procedure applied to obtain the experimental structure (73) generates too long hydrogen bonds between the base pairs. The last step of the structure determination was a constrained simulated annealing run using the AMBER10 program package (75). Such too long and too weak hydrogen bonds seem to be a general shortcoming of the AMBER force field, which will be the focus of a future publication. A similar behavior was seen for the borano-phosphate-modified DNA/RNA hybrid (PDB entry 2LAR, BMRB entry 17535) (76). In this case, even a weak correlation between the errors in the chemical shifts and the length of the hydrogen bond could be established (see supporting material).

### Non-canonical structures: the d(GCGAAGC) hairpin

**Figure 4.**
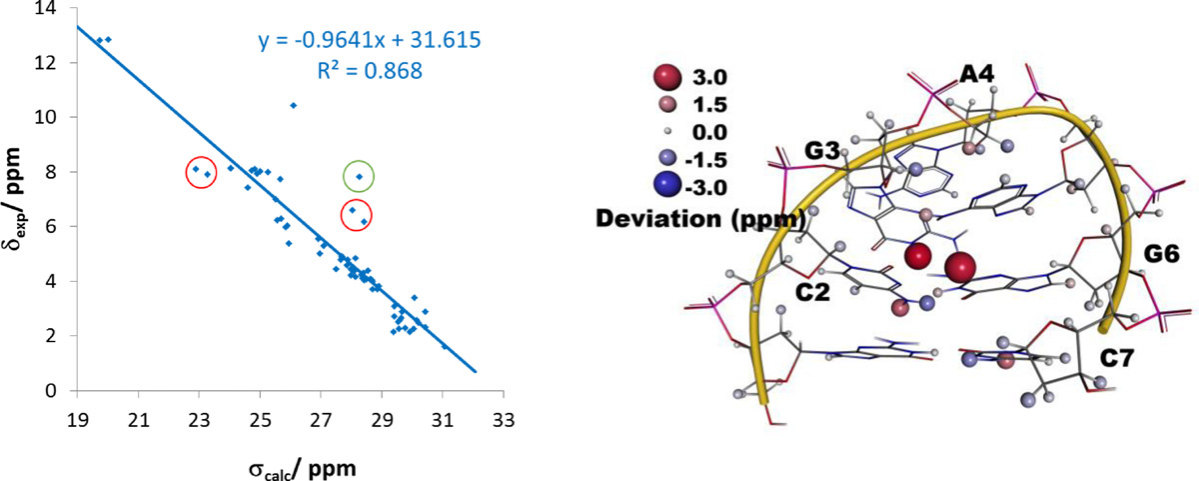
(Left) Correlation of the calculated isotropic chemical shielding with the experimental chemical shifts for the short d(GCGAAGC) hairpin structure (PDB entry 1KR8, BMRB entry 5282). (Right) Spatial distribution of the errors in the calculated ^1^H NMR chemical shifts.

As described in the introduction, empirical methods (46–51) are developed to predict NMR chemical shifts of DNA double helices. With the availability of more experimental data, we expect that these become as accurate as comparable approaches for proteins (1–7) being strong competitors of the approach proposed here because of their speed. The *ab initio* approach, however, has the advantage of being applicable to non-standard structural features for which the data basis for parameterization of empirical methods is unlikely to become available even within the next 10–20 years. We illustrate this with the example of a short d(GCGAAGC) hairpin structure (PDB entry 1KR8, BMRB entry 5282) (77). This structure was determined based on NOE-derived distances, torsion angles and residual dipolar couplings (RDCs). The overall very good correlation of the calculated and predicted chemical shifts (see Figure 4) underlines, on the one hand, the quality of the method, and, on the other hand, the extraordinary stability of this hairpin. A less stable structure showing global flexibility and/or partial unfolding would result in much higher errors due to the neglect of conformational averaging.

Only a couple of outliers derogate this good impression. The most significant errors in the calculations are obtained for H1 of G3 and H22 of G3 (green circle in Figure 4). Both polar protons are solvent exposed and, thus, probably form strong hydrogen bonds with the solvents. Neglecting the solvents leads again to an underestimation of the chemical shift. For the latter proton, there might be also another explanation for the deviation: H21 of this amino group forms a hydrogen bond to O2 of C2. The predicted value for this nucleus is very close to the experimental value of H22 and the correlation can be improved by assigning the experimental chemical shift to H21 (see Supplementary Figure S2 of the supporting information). However, please note that this is not proving a misassignment since the agreement for both chemical shifts should be improved when switching the experimental assignment but the experimental chemical shift of the other nucleus is not available. C2 and C7 show, however, chemical shift deviations of their amino groups where H41 and H42 is shifted to higher and lower field, respectively (red cycles in Figure 4). We also interchanged the experimental chemical shifts in these groups, but this resulted in a deterioration for all four nuclei (see also Supplementary Figure S2). Thus, in this case other reasons for the disagreement have to be found. While the overall results are, as mentioned above, incompatible with global structural changes, differences in local flexibility might influence the grade of accuracy of the predictions in the corresponding regions. Padrta *et al*. (77) discuss in the paper accompanying the structure that thermodynamic stability of the hairpin does not imply complete structural rigidity. They report sugar pucker wobbling in C7 and line broadening in the spectrum for A5. Indeed, the protons of C7 show higher deviations consistent with the discussed higher flexibility of the sugar moiety. Especially the high field shift of H41 of C7 can probably be explained by thermal fluctuations leading to an on average longer hydrogen bond to G1 compared with the experimental structure. In contrast, the calculated values of A5 do not show strong deviations from the experimental value. However, the chemical shifts of the sugar moiety of A4 are predicted poorer than the surrounding nuclei. These are very close to the aromatic system of A5 and, thus, are strongly influenced even by small motion of this group. Future calculations will show if ensemble averaging can describe these different flexibilities and can improve the agreement between calculation and experiment in more flexible regions.

### Nucleic acid/protein complexes: two lac repressor molecules bound to their natural operator O1

**Figure 5.**
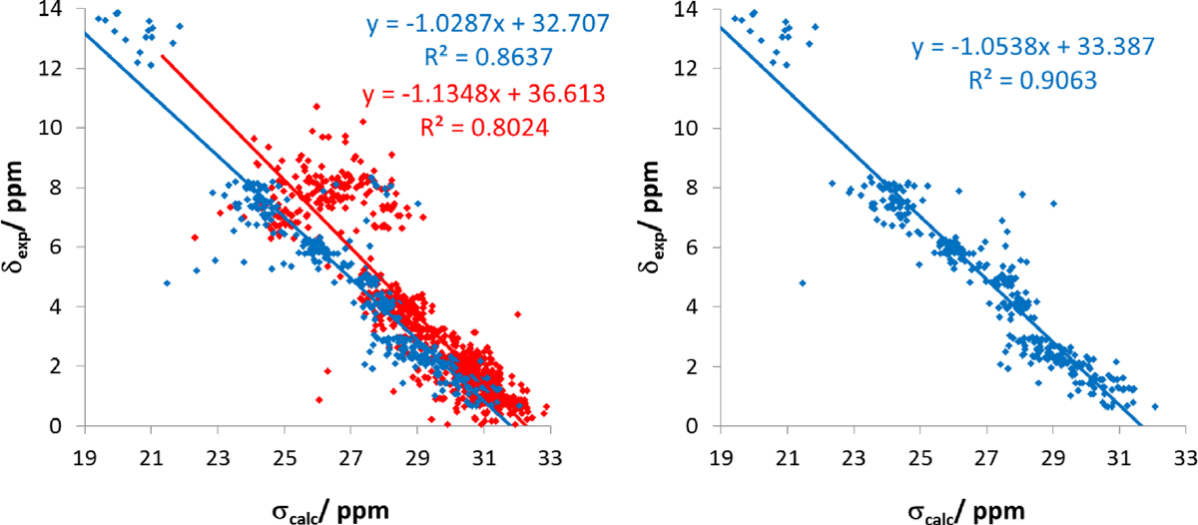
Correlations of the calculated isotropic chemical shielding with the experimental chemical shifts for the O1 operator DNA in the bound form with two lac repressor molecules (PDB entry 1L1M, BMRB entry 5345): (Left) the results for the DNA and the proteins are shown in blue and red, respectively. (Right) results for the DNA after interchanging the stereospecific assignment of amino groups in guanidine and cytosine.

The chemical shifts for the O1 operator DNA in the bound form with two lac repressor molecules (PDB entry 1L1M, BMRB entry 5345) (78) can also be predicted with acceptable accuracy considering the size of the system and the neglect of conformational averaging and solvents (see Figure 5). Especially, the non-polar protons in regions of the DNA only weakly disturbed by the binding are predicted with a similar accuracy as seen in the previous examples. Polar protons of the protein and solvent-exposed amino groups of the DNA bases are shifted to higher field due to missing explicit hydrogen bonds to the solvent as already discussed above and in previous publications (60,74). Similarly, hydrogen bond between base pairs are, as in the DNA duplex containing cidofovir discussed above, too long and the shifts are predicted too high field.

Beside these features common to all systems investigated so far, shifts to lower field are observed in the O1 operator DNA to an extent much larger than for other DNA systems. The chemical shielding of the H1 proton of dT15 of the first chain is predicted as 21.469 ppm, which is very close to the corresponding atoms in other thymidine bases. In contrast, the experimental chemical shift is given as only 4.791 ppm, which is very different to the usual values of around 13 ppm for this atom type found in the BMRB. There is no obvious reason for this extreme chemical shift. The deviation from the normal B-DNA structure is here not larger than in other parts of the structure and the interactions with the surrounding protein groups are expected to cause chemical shift perturbations in the normal range of at maximum 1 to 2 ppm only. Therefore, we speculate that this is a typo in the experimental data file. The other larger deviations to lower field belong to amino groups of guanidine and cytosine residues and appear always in pairs of one too high-field (H21 and H41 in G and C, respectively) and one too low-field (H22 and H42, respectively) shifted proton. As in the case of the hairpin described above this might be caused by a problem in the stereospecific assignment of the amino group. In contrast to 1KR8, interchanging the chemical shifts for these groups in the experimental data file resulted in a strongly improved correlation and errors in the predictions of the same size as for the surrounding protons (see Figure 5 and supporting information for a visualization of the improved accuracy mapped onto the molecular structure). One additional indication of this potential misassignment comes from the comparison to other BMRB entries. In the corresponding files to PDB entries 1AHD, 1KR8, 2KXM and 2L8P discussed here or in the supporting information, the larger chemical shifts are assigned to H21 ($\delta _{{\rm H}21} {>} 7\,{\rm ppm}$ and $\delta _{{\rm H}22} {<} 7\,{\rm ppm}$) and H41 ($\delta _{{\rm H}41} {>} 7\,{\rm ppm}$ and $\delta _{{\rm H}42} {<} 7\,{\rm ppm}$) in G and C, respectively. In the BMRB entry of the O1 operator DNA, the exact opposite is found. One special case is DG8, in which the same experimental chemical shift is assigned to H21 and H22, e.g. the stereospecificity was not taken into account at the stage of chemical shift assignment. Since the value is with 7.450 ppm larger than 7, we assigned it to H21 in our optimized assignment and treated H22 as unassigned. In many instances, stereospecific assignments are handled in a floating fashion during structure refinement potentially without properly updating the BMRB entry. If this is the case here cannot be deducted from the publication (78).

**Figure 6.**
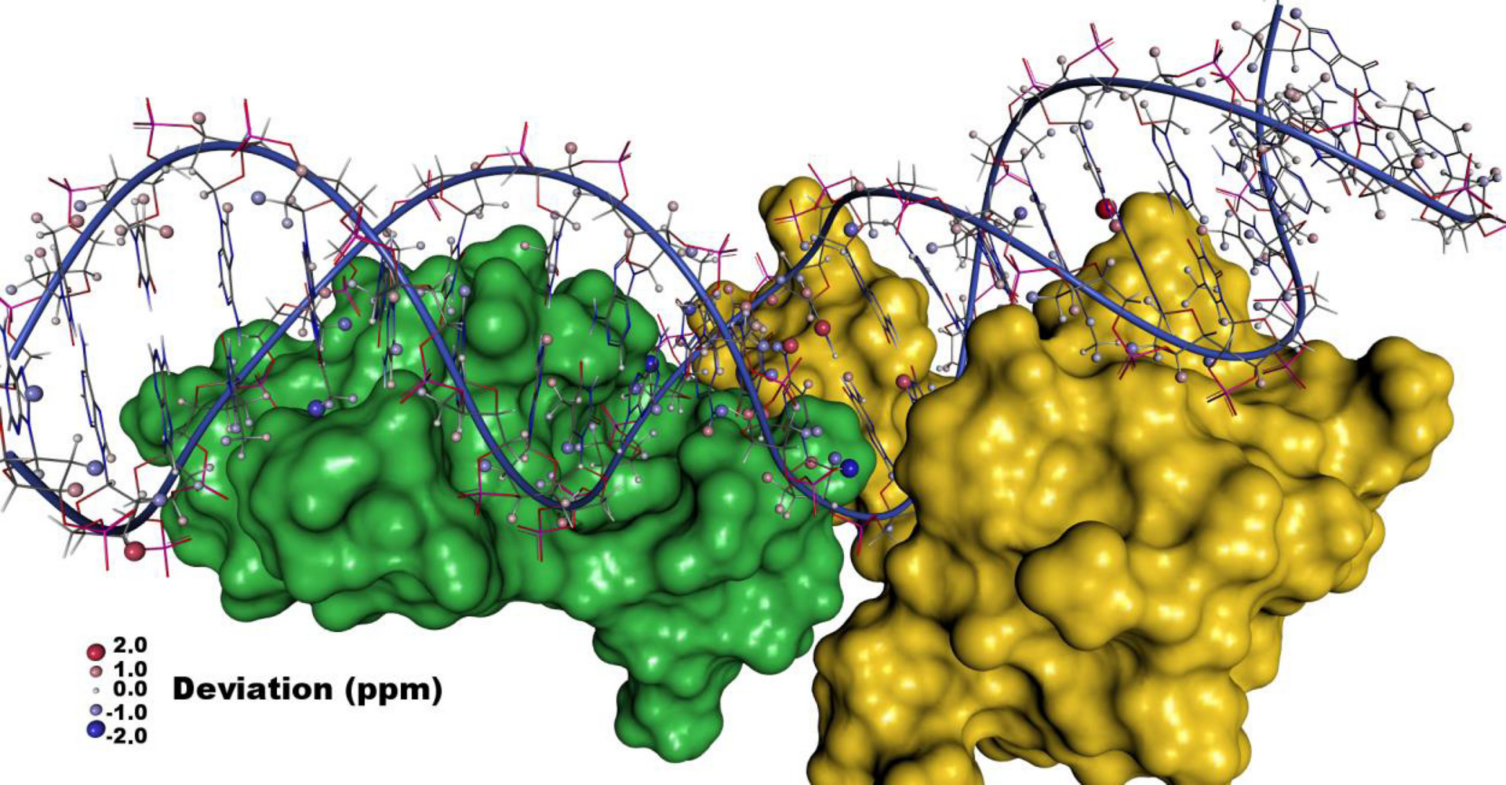
Spatial distribution of the errors in the calculated ^1^H NMR chemical shifts in the complex of two lac repressor molecules (yellow and green surface) bound to their natural operator O1 (PDB entry 1L1M, BMRB entry 5345).

The spatial distribution of the remaining errors after adaptation of the stereospecific assignments and removing the nuclei involved in the suboptimal hydrogen bonds of the base pairs as well as H1 of DT15 and H22 of DG8 is presented in Figure 6. Large parts of the system including the interaction sites of O1 operator DNA with one lac repressor protein shows small deviations only, which demonstrates that the influence of the protein can be modeled with reasonable accuracy. In the center of the figure, however, an accumulation of errors can be seen. Here, the operator interacts with both lac repressor proteins simultaneously resulting in a strong deformation of the double helix. Higher flexibility is, on the one hand, expected when the DNA is forced into such a less favorable conformation. On the other hand, modeling of such non-canonical structures is probably also accompanied by larger structural uncertainties due to suboptimal force fields. If the first or the latter reason is mainly causing the reduced accuracy has to be determined in future investigations.

### G-Quadruplexes: the hTERT promoter sequence

The formation of G-quadruplexes inhibits the activity of the telomerase hTERT and the development of specific telomeric quadruplex-stabilizing ligands could lead to anticancer drugs (79). This is the case since human telomeric repeats are highly polymorphic being able to adopt at least five different G-quadruplex folding topologies under different experimental conditions (80,81). Lim *et al*. (82) showed that one particular G-rich sequence of the hTERT promoter coexists in two G-quadruplex conformations in solutions containing 100-mM potassium ions. By sequence modification, i.e. guanine-to-inosine substitutions, each conformation could be stabilized and analyzed independently by NMR and CD spectroscopy. The resulting structures showing a (3 + 1) and an all-parallel-stranded G-quadruplex are deposited as entries 2KZD and 2KZE in the PDB, respectively.

**Figure 7.**
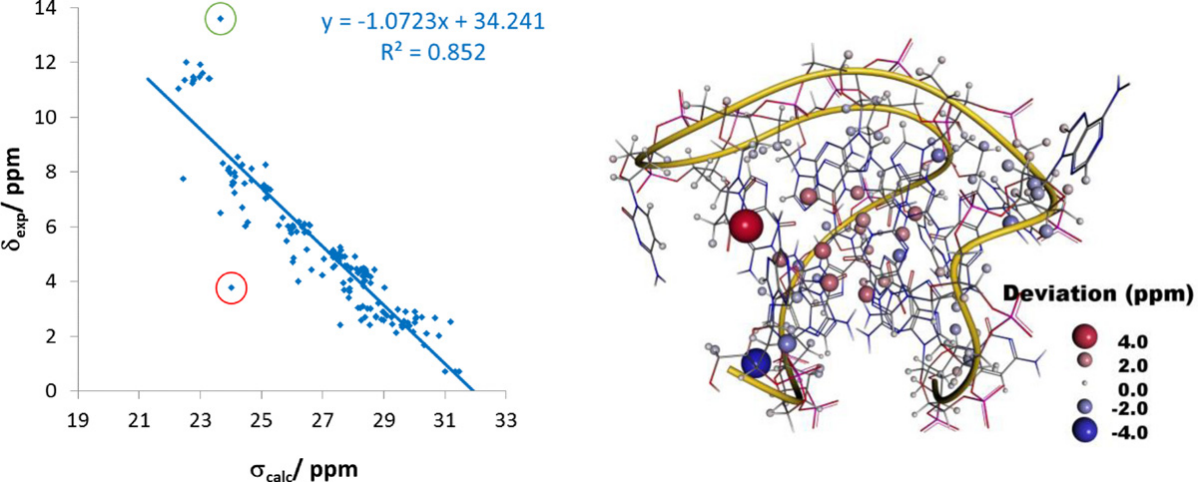
(Left) Correlation of the calculated isotropic chemical shielding averaged over all 10 NMR models with the experimental chemical shifts for the (3 + 1) G-quadruplex formed by the hTERT promoter sequence (PDB entry 2KZD). The two largest errors in the nucleotides A1 and T12 are marked with a red and green circle, respectively. (Right) Spatial distribution of errors in the calculated ^1^H NMR chemical shifts.

**Figure 8.**
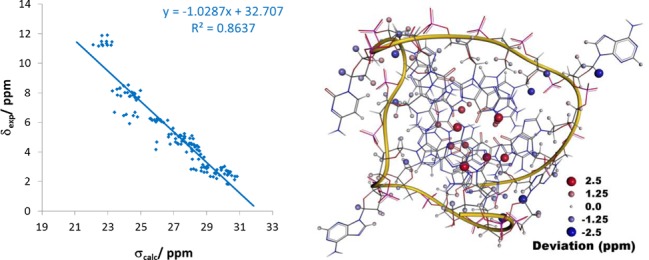
(Left) Correlation of the calculated isotropic chemical shielding averaged over all 10 NMR models with the experimental chemical shifts for the all-parallel-stranded G-quadruplex formed by the hTERT promoter sequence (PDB entry 2KZE). (Right) Spatial distribution of errors in the calculated ^1^H NMR chemical shifts.

**Figure 9.**
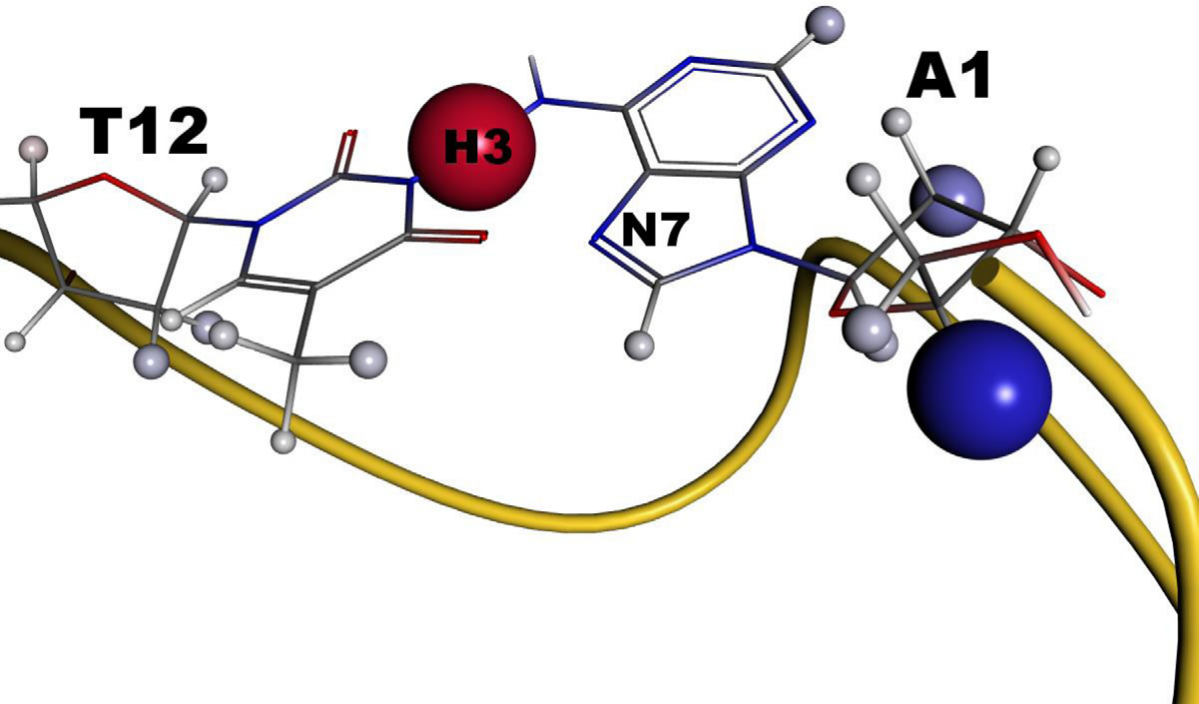
Nucleotides showing the largest deviations between the calculated and experimental chemical shifts in the the (3 + 1) G-quadruplex formed by the hTERT promoter sequence (PDB entry 2KZD). By a small rearrangement of the terminal nucleotide A1, the large errors in the backbone could be removed and a hydrogen bond to T12 could be formed also removing the large error in this nucleotide.

We predicted the NMR chemical shifts of all 10 models of each structure and compared these to the experimental results provided by Anh Tuan Phan (private communication). When looking at the summarized results in Table 1, the large mean absolute errors and the large dependence of these errors on the used model become evident. These are, however, neither a sign of a wrong structure calculation nor of a failure of the quantum mechanical chemical shift predictions. The spatial distribution of errors (see Supplementary Figures S4 and S5 of the supporting information) show lower deviations in the central regions in analogy to the deviations seen in other examples of this study. This includes the poor prediction of protons involved in hydrogen bonds. Much larger errors of up to 6 ppm are only calculated in case of very flexible parts of the G-quadruplexes. Lim *et al*. named some specific regions, e.g. the three-purine loop I5-A6-G7 in 2KZD, where the structure is less well defined, probably reflecting the presence of multiple conformations within these regions (82). Therefore, it is clear that the prediction based on one single structure will fail here and conformational averaging is essential. As a first approximation for doing so, we averaged the chemical shifts over all 10 experimental models. The mean absolute errors for these averaged chemical shifts are for both structures (2KZD: MAD = 0.674, 2KZE: MAD = 0.586) very close to the best single model of the corresponding ensembles. Especially the errors in the flexible regions are dramatically reduced. However, it should be kept in mind here that a standard NMR ensemble need not perfectly reflect the real distribution of conformers especially because during the generation of NOE constraints short distances tend to be over-represented. Even more improved agreement should be obtainable using conformational sampling by MD simulations starting from the NMR ensembles. For 2KZE, no chemical shift is predicted with an error larger than 2.6 ppm and it is interesting to note that the largest deviation is caused by the suboptimal description of a hydrogen bond and not by insufficient sampling of the flexible parts (see Figure 7). In contrast, 2KZD shows two nucleotides with very large errors even after averaging (see Figure 8). The most prominent nucleus is H3 of T12, which is predicted with an error of 4.991 ppm. Its experimental value is 13.604 ppm indicative of a strong hydrogen bond, which was already discussed in the original paper (82). They proposed the formation of a reverse Hoogsteen base pair between T12 and A1, which is additionally supported by NOE signal to H8 of A1. Such bond is, however, not present in any of the experimental models and it can also not be formed with the solvent due to the proximity of A1 (see Figure 9). Nevertheless, N7 of A1 could re-orientate to allow for the formation of a very favorable hydrogen-bond by only small structural variations of the terminus. This re-orientation could probably also help to remove the large errors in the backbone of A1. MD simulations are on their way to verify the assumed conformational changes.

One possible goal of the chemical shift prediction is the identification of the correct structure of a system out of a number of decoys. To see if this is possible for the hTERT promoter, we compared the predicted chemical shifts of the 2KZD models with the experimental values of 2KZE and vice versa. For both sets of chemical shifts, the MAD predicted on the correct ensemble is smaller than the one for the decoy ensemble (2KZD: MAD_correct_ = 0.674 ppm, MAD_decoy_ = 0.722 ppm; 2KZE: MAD_correct_ = 0.586 ppm, MAD_decoy_ = 0.692 ppm). Even if this can be regarded as success, the results have to be interpreted with caution. For such highly flexible structures, the results extremely depend on the chosen conformations. If looking at single conformations, many correct and decoy structures have almost the same MAD and the MAD of the best decoy is more than 0.1 ppm better than of the worst correct structure. Thus, sufficient conformational sampling preferable using improved force fields and including explicit solvents should be performed before structure identification based on chemical shift prediction is attempted.

## CONCLUSION

The results presented here and in the supporting information clearly show that quantum chemical fragmentation approaches are able to predict NMR chemical shifts of canonical and non-canonical structures of nucleic acids with good accuracy. MAD of 0.4–0.6 ppm are obtained for all system except the very flexible G-quadruplexes. This compares very favorable to the results obtained on polypeptides and proteins. For the HA2 domain of the influenza virus glycoprotein hemagglutinin, e.g. MADs of 0.84 and 0.45 ppm result for all and non-polar protons, respectively (59). The remaining deviations between experiment and calculation can be assigned to structural flexibility and/or, as in proteins (60,74), to the incorrect description of the interactions with the solvent, which is only described by an electrostatic continuum in our present calculations. It has been shown that these effects can be correctly described in proteins by conformational averaging and explicit solvents molecules using molecular dynamics simulation and multiple quantum chemical calculations of snapshots taken from these MD trajectories (60). In this way, temperature effects on experimental chemical shift values can also be taken into account by appropriately choosing the simulation conditions in contrast to the single-conformation calculations performed here, which are effectively done at 0 K. Currently the shortcomings of state-of-the-art force fields in correctly representing H-bonding distances (geometries), however, certainly limit the expectable accuracy to predict temperature effects on the chemical shifts of imino protons as seen e.g. in (83). Similar calculations for nucleic acids are ongoing in our group. One new effect is the relatively large deviation of protons forming hydrogen bonds between base pairs seen in almost all systems. This can probably be attributed to too long and too weak hydrogen bonds modeled by state-of-the-art force fields used in the structure determination process based on NMR data (mainly NOE distance constraints). Calculations are on their way to further prove this hypothesis. Overall, results of this systematic investigation again justify and motivate the use of NMR chemical shifts as a probe for further DNA/RNA force field developments especially with the very high sensitivity of quantum chemical methods.

Beside these common features, very specific problems can be identified in different systems. In the O1 operator DNA in complex with two lac repressor molecules a typo in the experimental assignment file could be identified. Additionally, flipping the stereospecific assignment of some amino groups of guanidine and cytosine residues resulted in an improved agreement between experiment and prediction. Unfortunately, it is not possible to extract from the literature if such a flipping was also performed in the course of the structure determination. Such misasignments show up as single outliers in an otherwise reasonably predicted region of the structure. Additionally, high flexibility and, in this way, high uncertainty in the structures or even larger problems in the structure determination process could be identified by deviations of larger regions when comparing experimental and theoretical chemical shifts. This can be nicely seen with the G-quadruplex structures, which show large variations between the models in parts of the structures which was explained with the presence of multiple conformations in these regions (82). Predictions based on one single model yield very large errors in these flexible loops which can partly be removed by averaging over all 10 models. The remaining large errors in the (3 + 1) conformation can be attributed to a slightly wrong orientation of the terminal nucleotide A1 due to which the hydrogen bond to T12 cannot be formed. One additional example of this kind is probably the riboswitch N1 with bound ribostamycin discussed in the supporting information, where the deviations cannot be explained exclusively by the failure of the theoretical method.

These results together with the publications cited in the introduction highlight the high potential of quantum chemical calculation of NMR chemical shifts for a number of applications in nucleic acid research. Structure evaluation as well as force-field optimization are just two of these primarily serving very different scientific communities. The advent of even faster computers might allow for including conformational averaging and explicit solvents in the near future opening the route to significantly improved chemical shift predictions, automated assignment procedures and even 3D structure prediction. In addition to the systems presented here, many other very interesting DNA/RNA structures like triplexes, DNA duplexes with mispairs and complex RNAs exist and it would be highly valuable to perform chemical shift calculations on those. Unfortunately, this was not possible in the study described here due to limitations of computer resources and availability of experimental data. Even with the fragment-based approach, such calculations are far from trivial and high-performance computer resources are needed. Nevertheless, we will continue such calculations on system of particular interest and gather all these results in a database. We hope that this will provide the information needed to extend and improve empirical methods such that they could be used as part of a refinement process using the chemical shifts directly.

## SUPPLEMENTARY DATA

Supplementary Data are available at NAR Online.

SUPPLEMENTARY DATA

## ACCESSION NUMBERS

PDB IDs: 2KZD, 2L8P, 1KR8, 1L1M, 2LAR, 1AHD, 2KKM and 2KZE.
