# Supplementary material for: Accurate ab initio prediction of NMR chemical shifts of nucleic acids and nucleic acids/protein complexes
Source: Nucleic Acids Res. 2014 Nov 17;42(22):e173. doi: 10.1093/nar/gku1006 (PMC4267612; doi:10.1093/nar/gku1006)
Supplement: SUPPLEMENTARY DATA [file supp_42_22_e173__index.html]

Accurate ab initio prediction of NMR chemical shifts of nucleic acids and nucleic acids/protein complexes — Accurate ab initio prediction of NMR chemical shifts of nucleic acids and nucleic acids/protein complexes — SUPPLEMENTARY DATA 

# Accurate *ab initio* prediction of NMR chemical shifts of nucleic acids and nucleic acids/protein complexes

## SUPPLEMENTARY DATA

**Files in this Data Supplement:**

- SUPPLEMENTARY DATA
