# Supplementary material for: Accurate ab initio prediction of NMR chemical shifts of nucleic acids and nucleic acids/protein complexes
Source: Nucleic Acids Res. 2014 Nov 17;42(22):e173. doi: 10.1093/nar/gku1006 (PMC4267612; doi:10.1093/nar/gku1006)
Supplement: SUPPLEMENTARY DATA [file supp_gku1006_NMRADMA5_Supporting_20140922.pdf]

# Supporting Information:

# Accurate *Ab Initio* Prediction of NMR Chemical Shifts of Nucleic Acids and Nucleic Acids / Protein Complexes

Andrea Victora[1], Heiko M. Möller[1,2] and Thomas E. Exner[3,*]

[1] Department of Chemistry and Zukunftskolleg, Universität Konstanz, 78457 Konstanz, Germany
[2] Institute of Chemistry, University of Potsdam, Karl-Liebknecht-Str. 24-25, 14476 Potsdam OT Golm, Germany
[3] Institute of Pharmacy, Eberhard Karls Universität Tübingen, Auf der Morgenstelle 8, 72076 Tübingen, Germany

**SECOND CHAIN OF DNA DUPLEX CONTAINING CIDOFLOVIR**

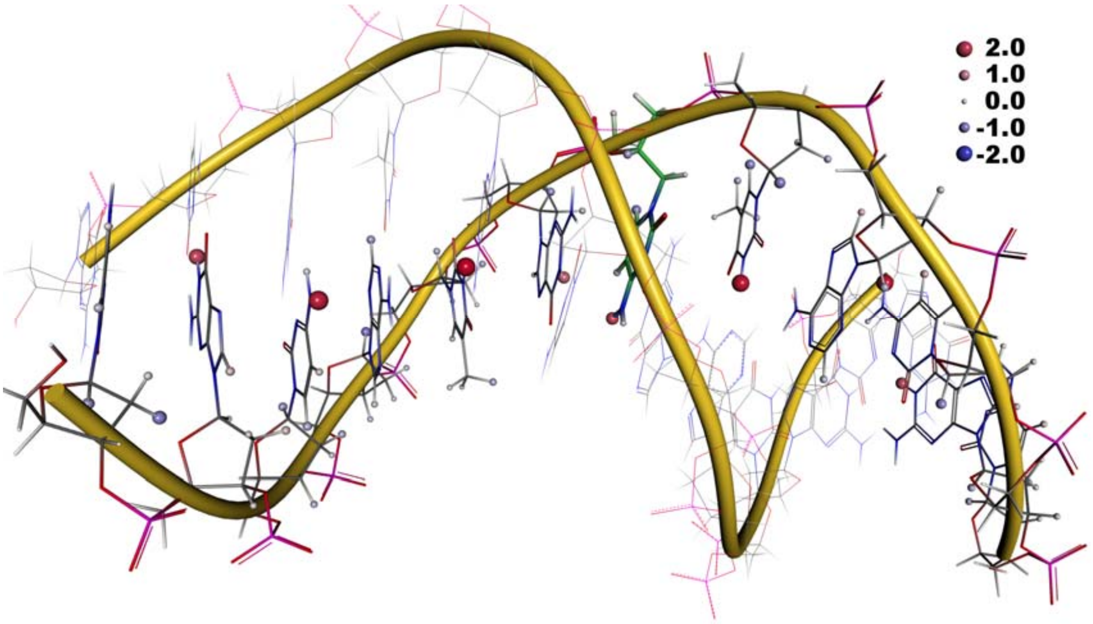


**Figure S1**: Spatial distribution of the errors in the calculated $^{1}H$ NMR chemical shifts for the second chain of a DNA duplex containing the potent anti-poxvirus agent cidoflovir (PDB entry 2L8P): Cidoflovir is marked with green carbon atoms. The nuclei of the two groups, which are predicted high-field-shifted compared to the experiment, are clearly seen as the large red spheres with errors larger than 1-2 ppm.

**INTERCHANGING OF STEREOSPECIFIC ASSIGNMENT IN A SHORT HAIRPIN STRUCTURE**

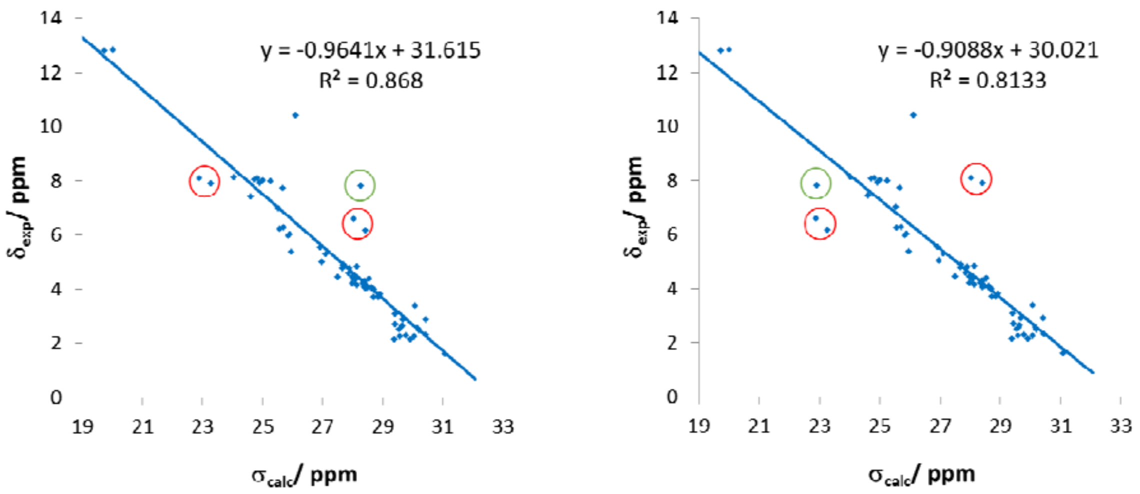


**Figure S2**: (left) Correlations of the calculated isotropic chemical shielding with the experimental chemical shifts for the short d(GCGAAGC) hairpin structure (PDB entry 1KR8, BMRB entry 5282) after interchanging the stereospecific chemical shifts of amino groups in C2, G6, and C7. (right) Correlation using the original assignment is shown again for easier comparison. The interchange leads to an overall worsening of the agreement.

**INTERCHANGING OF STEREOSPECIFIC ASSIGNMENT IN O1 OPERATOR DNA**

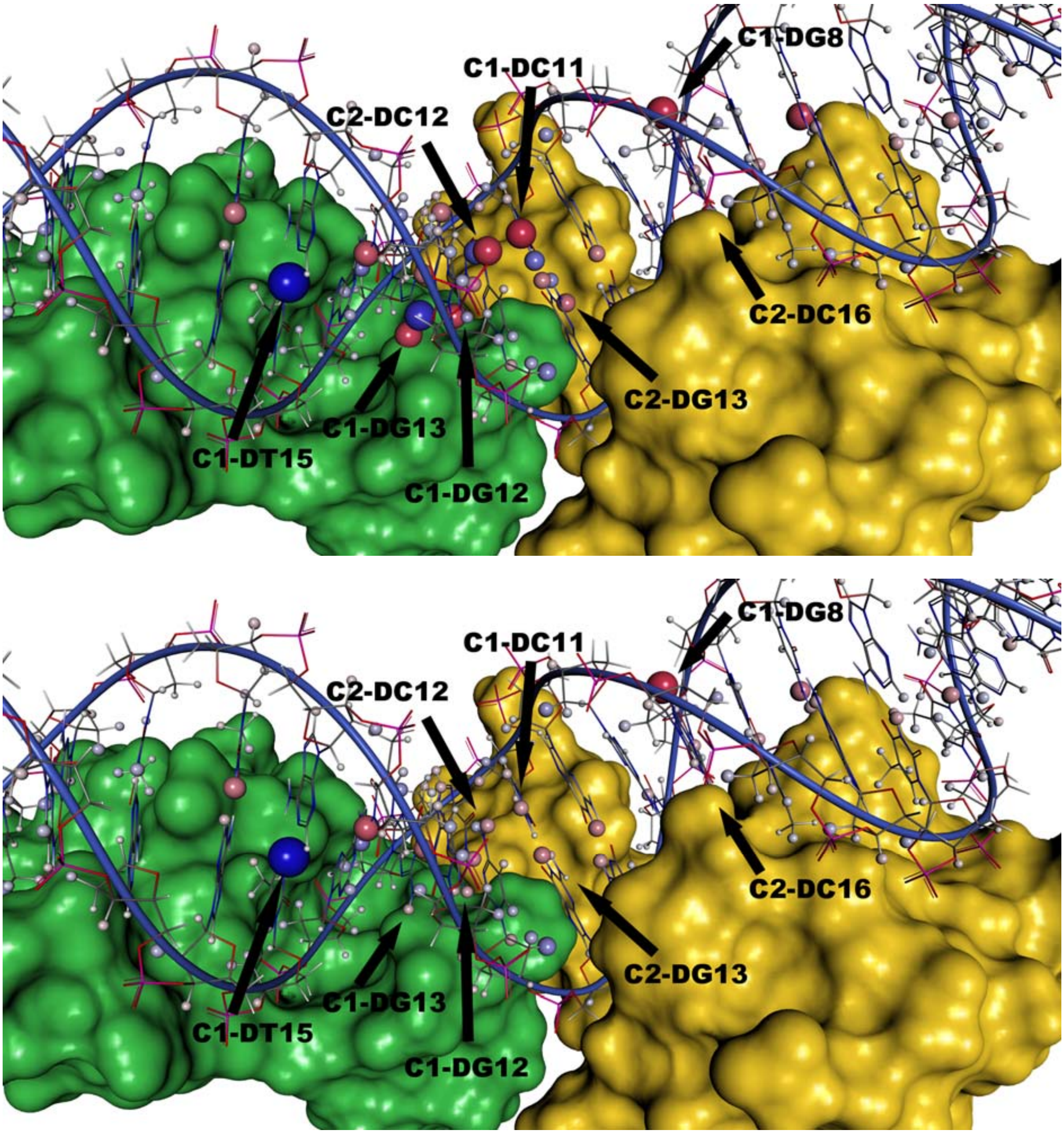


**Figure S3:** Spatial distribution of the errors in the calculated $^{1}$H NMR chemical shifts in the complex of two lac repressor molecules (yellow and green surface) bound to their natural operator O1 (PDB entry 1L1M, BMRB entry 5345): (upper part) original assignment stored in the BMRB, (lower part) optimized assignment by interchanging the stereospecific chemical shifts of amino groups in guanidine and cytosine. The improvements for all bases except DG8, for which no stereospecific assignment is available, can clearly be seen.

**COMPARISON OF DIFFENENT MODELS OF THE QUADRUPLEX FORMED BY HTERT PROMOTER SEQUENCE**

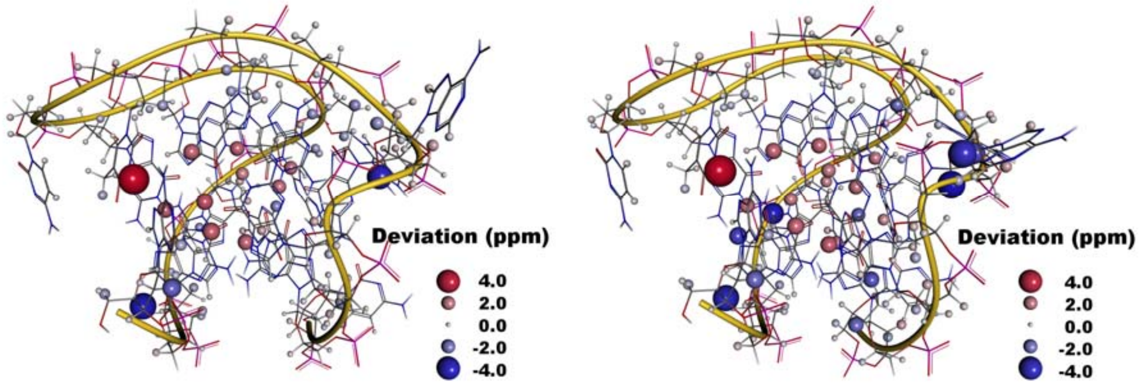


**Figure S4:** Spatial distribution of the errors in the calculated $^1$H NMR chemical shifts of the (3+1) G-quadruplex formed by hTERT promoter sequence (PDB entry 2KZD) obtained with the model with the best (model 1, MAE = 0.653 ppm) and worst (model 2, MAE = 0.808 ppm) agreement to experiment is shown on the left and right, respectively.

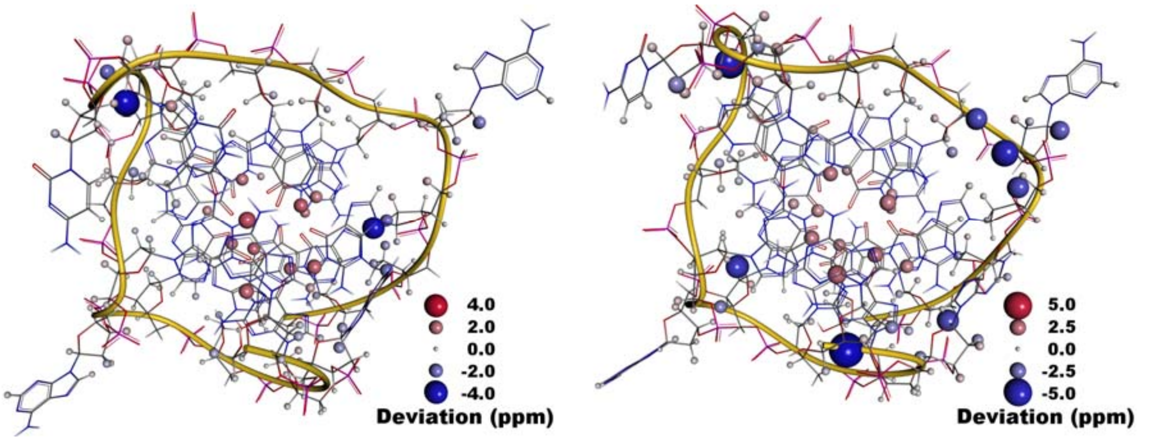


**Figure S5:** Spatial distribution of the errors in the calculated $^1$H NMR chemical shifts of the all-parallel-stranded G-quadruplex formed by hTERT promoter sequence (PDB entry 2KZD) obtained with the model with the best (model 2, MAE = 0.560 ppm) and worst (model 2, MAE = 0.805 ppm) agreement to experiment is shown on the left and right, respectively.

## ADDITIONAL EXAMPLES

### Double Helices: Small Palindromic DNA Duplexes

Two small palindromic double-helical DNA structures were analyzed. The first is the d(CGTACG)$_2$ sequence (PDB entry 1K2K, BMRB entry 5339) investigated by Lam and Ip (1) at low temperature. The second sequence d(TGATCA)$_2$ (PDB entry 1SY8, BMRB entry 6186) was investigated by Barthwal et al. (2) as a model system, which includes the 5'd-TpG 3' element involved in the regulation of gene expression as well as targeted by several intercalating anticancer drugs. The correlation of the calculated isotropic NMR chemical shieldings and the experimental chemical shifts are shown in Figure S6. For 1SY8, we will only discuss the 1$^{st}$ of the 10 model structures stored in the PDB file. The results obtained with the others show very similar behavior (data not shown). Very good correlation coefficients and slopes close to the optimal value of -1 are obtained for both systems. The slight advantage of the 1K2K structure compared to 1SY8 can probable be explained by the low temperature of the measurement and the resulting reduced thermal motion at these temperatures. In the original publication, structures at 5, 10, and 15°C were compared. Our calculations are based on the 15°C structure. For further analysis of the errors in the calculations, we converted the chemical shieldings into calculated chemical shifts as described in the Material and Methods section. For the chemical shielding of the standard values of 32.29 and 32.15 ppm were obtained for 1K2K and 1SY8, respectively, reasonably close to the quantum chemical shielding of 32.24 ppm determined for TMS with the same level of theory / basis set combination. When looking at the spatial distribution of the errors in 1K2K (Figure S7), a concentration of the errors at the termini becomes evident. This can be explained by the experimental findings that at higher temperature the stacking interactions among the central base pairs improve facilitated by fraying of the termini (1). Larger flexibility leads to larger uncertainties in the calculated chemical shifts as shown by the large improvements, which can be obtained when including conformational averaging as described in our previous publications (3;4). In 1SY8, the errors are distributed more evenly (see also Figure S7), which agrees with the experimentally confirmed larger flexibility of the complete structure especially of the sugar-phosphate backbone with rapid inter-conversion between two different conformations at the TpG/CpA base pairs (2). To cover this, a multi-conformation approach is essential (3;4). There is, however, an additional explanation for the large errors at the sugar moiety of T1. The terminating phosphate group is not correctly represented in the PDB file, *i.e.* one oxygen atom is missing and the remaining three remaining substituents and the phosphorus are arranged in a plane, which has a significant influence on the calculated chemical shifts in close proximity.

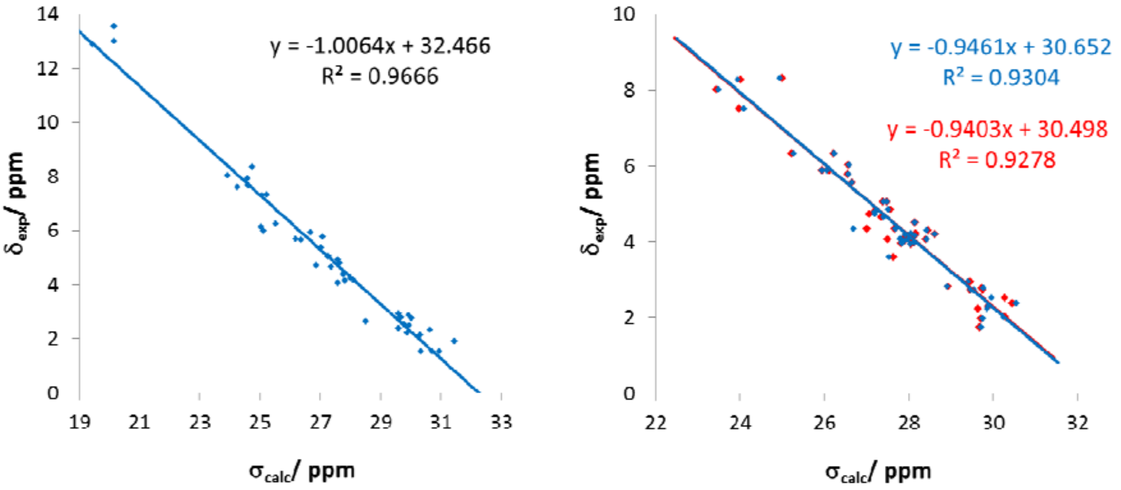


**Figure S6:** Correlations of the calculated isotropic chemical shielding with the experimental chemical shifts for the two short double-helical DNA structures d(CGTACG)$_2$ (PDB entry 1K2K, left) and d(TGATCA)$_2$ (PDB entry 1SY8, right). For 1SY8, results for the 1$^{st}$ structure (red) and the average of all 10 structures (blue) show almost no difference in the parameters of the correlation line.

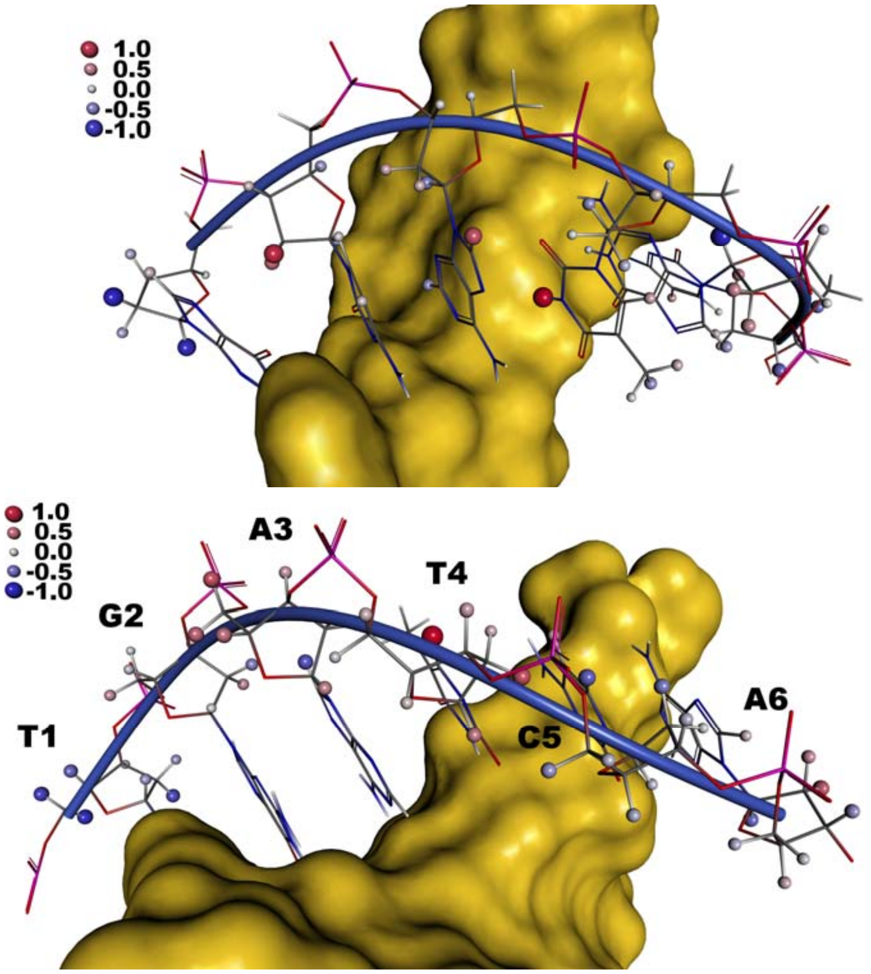


**Figure S7:** Spatial distribution of the errors in the calculated $^1$H NMR chemical shifts for the two short palindromic double-helical DNA structures d(CGTACG)$_2$ (PDB entry 1K2K, upper) and d(TGATCA)$_2$ (PDB entry 1SY8, lower): The values are coded by the color and size of the spheres representing the atoms (darker color and larger spheres correspond to larger error).

**Double Helices: Borano-Phophate Modified DNA/RNA Hybrid**

Johnson at al. (5) studied the structures of borano-phosphate-modified nucleic acids and their acceptance as substrates by RNase H1. More specifically, they solved two structures of modified DNA/RNA hybrids using a large number of experimental constraints including RDCs and backbone torsion angles and found helical properties between those of an A and B helix. We predicted the NMR chemical shifts for one of these (central stereo specific Rp borano phosphate linkage, PDB entry 2LAR, BMRB entry 17535). The results are very comparable to 2L8P with excellent overall correlation but larger errors for the protons involved in the central hydrogen bond between the base pairs (see Figure S8). Removing those from the correlation results in an almost perfect slope of -0.983. To follow our hypothesis of too long and too weak hydrogen bonds produced by the modelling method during the structure-determination procedure, we plot the error in the predictions versus the distance of the central hydrogen bond (see Figure S9). Even if we expected some correlation, the almost perfect linear correlation was a surprise. Only one of the termini (U18) shows a larger deviation from the correlation line. A somewhat larger distance and, thus, smaller relative error is expected due to fraying of the termini. This definitely does not imply that one should scale the distances linearly to the errors to get to the “real” system. It should, however, be checked if distance constraints between the base pairs or improved force-field parameters forcing them closer together could help to improve the predictions of these and other nuclei close in space.

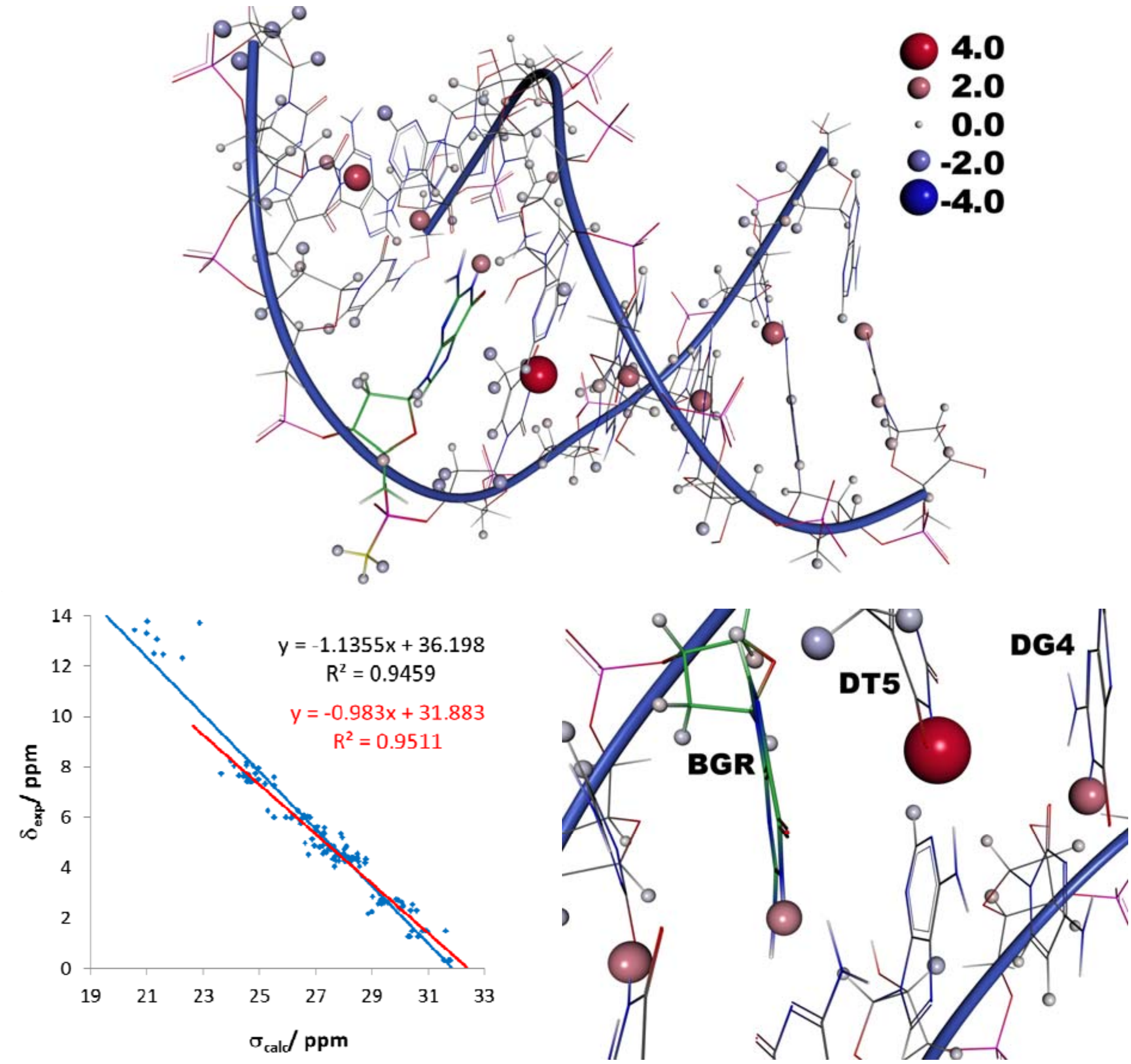


**Figure S8:** (upper) Spatial distribution of the errors in the calculated $^{1}$H NMR chemical shifts for the borano-phophate modified DNA/RNA hybrid (PDB entry 2LAR, BMRB entry 17535). (lower left) Correlations of the calculated isotropic chemical shielding with the experimental chemical shifts The correlation coefficient and especially the slope can be improved by neglecting the protons involved in the central hydrogen bond of the base pairs (red line). (lower right) Central part around the borano phosphate.

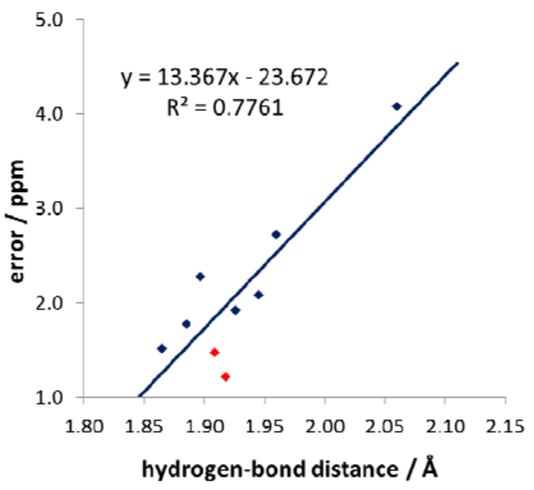


**Figure S9:** Correlation of the errors in the predicted NMR chemical shifts versus the length of the central hydrogen-bond between the base pairs. The two terminal base (G10 and U18) are marked in red.

**Non-Canonical Structures: Riboswitch N1 with Bound Ribostamycin**

We now look at the very important class of riboswitches. The at-the-time-of-writing smallest riboswitch N1 functional in vivo was investigated by NMR spectroscopy (6). It contains only 27 nucleotides in a bulged hairpin secondary structure and responds to the antibiotics neomycin B and ribostamycin. In Figure S10 the predicted chemical shifts of N1 with bound ribostamycin are correlated with the experimental observed ones based on the first model of PDB entry 2KXM (BMRB entry 16609). These are not as good as seen for the other examples discussed up to now. Whether this is caused by larger flexibility or by problems in the structure determination due to the unusual structural features has to be investigated in the future. One argument for the latter are the large errors around the ligand also shown in Figure S10

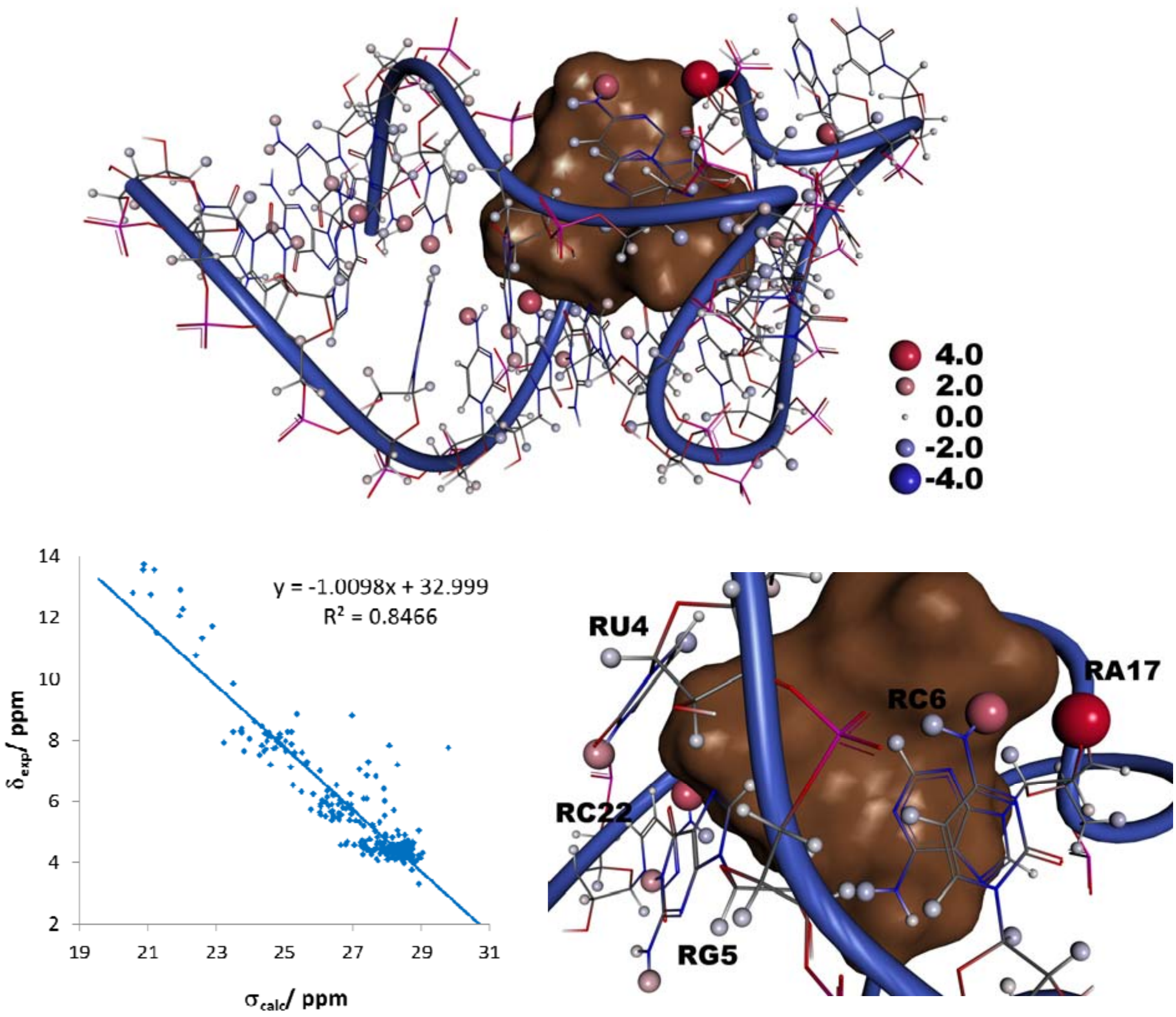


**Figure S10:** (upper) Spatial distribution of the errors in the calculated $^{1}$H NMR chemical shifts for the riboswitch N1 (PDB entry 2KXM, BMRB entry 16609). (lower left) Correlations of the calculated isotropic chemical shielding with the experimental chemical shifts. (lower right) Region around the bound neomycin B (brown surface).

**Nucleic Acid / Protein Complexes: Antennapedia Homeodomain-DNA Complex**

One of the first DNA-protein complex solved by NMR spectroscopy is the Antennapedia homeodomain-DNA complex. It was published in 1993 by the Wüthrich group (7). We again used the first model to predict the chemical shifts. The quality of the results of the same high accuracy as for the double helix structures described above (see Figure S11). Beside the high-field shift of polar protons forming hydrogen bonds in the base pairs, some protons of the bases forming hydrogen bonds to the solvent are also predicted with relative large errors. Other than that, no large deviations even at the interface to the protein are seen (Figure S11). This speaks, on the one hand, for the excellent work done by Billeter et al. (7) more than 20 years ago and, on the other hand, the general applicability of our chemical shift calculations. In 1998, Fraenkel and Pabo compared the NMR structure to the newly determined X-ray structure of the same complex (8). They identified larger

differences in the interactions of the three amino acids ARG43, GLN50, and ASN 51 with A20, C7, and A19/A20, respectively. Since the predictions of the chemical shifts in these regions do not deviate more than in other regions, our calculations support the original NMR solution structure or at least do not disprove it. Calculations are on their way to analyze the agreement between the X-ray structure and the NMR chemical shift. If these show large deviations in the regions around the three amino acids, the structural differences have to be caused by crystal packing effects.

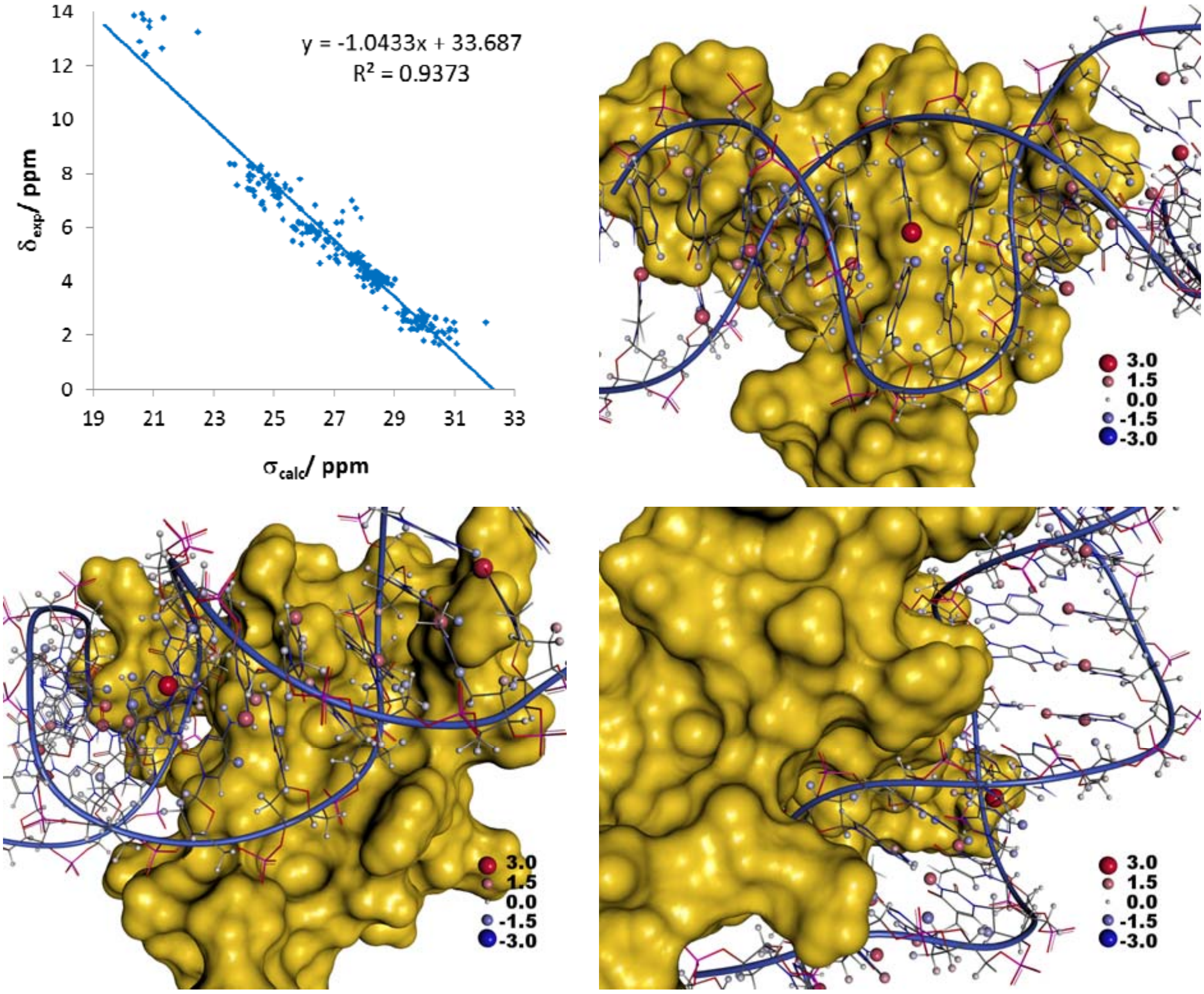


**Figure S11:** (upper left) Correlations of the calculated isotropic chemical shielding with the experimental chemical shifts for the Antennapedia homeodomain-DNA complex (PDB entry 1AHD, BMRB entry 4104). (upper right, lower left, and lower right) Three different views of the spatial distribution of the errors in the calculated $^1$H NMR chemical shifts close to the protein (yellow surface).

1. Lam,S.L. and Ip,L.N. (2002) Low Temperature Solution Structures and Base Pair Stacking of Double Helical d(CGTACG)$_2$ *J.Biomol.Struct.Dyn.*, **19**, 907-917.

2. Barthwal,R., Awasthi,P., Monica, Kaur,M., Sharma,U., Srivastava,N., Barthwal,S.K. and Govil,G. (2004) Structure of DNA sequence d-TGATCA by two-dimensional nuclear magnetic resonance spectroscopy and restrained molecular dynamics *J.Struct.Biol.*, **148**, 34-50.

3. Exner,T.E., Frank,A., Onila,I. and Möller,H.M. (2012) Toward the Quantum Chemical Calculation of NMR Chemical Shifts of Proteins. 3. Conformational Sampling and Explicit Solvents Model *J.Chem.Theory Comput.*, **8**, 4818-4827.

4. Dracinsky,M., Möller,H.M. and Exner,T.E. (2013) Conformational Sampling by Ab Initio Molecular Dynamics Simulations Improves NMR Chemical Shift Predictions *J.Chem.Theory Comput.*, **9**, 3806-3815.

5. Johnson,C.N., Spring,A.M., Sergueev,D., Shaw,B.R. and Germann,M.W. (2011) Structural Basis of the RNase H1 Activity on Stereo Regular Borano Phosphonate DNA/RNA Hybrids *Biochemistry*, **50**, 3903-3912.

6. Duchardt-Ferner,E., Weigand,J.E., Ohlenschläger,O., Schmidtke,S.R., Suess,B. and Wöhnert,J. (2010) Highly Modular Structure and Ligand Binding by Conformational Capture in a Minimalistic Riboswitch *Angew.Chem.Int.Ed.*, **49**, 6216-6219.

7. Billeter,M., Qian,Y.Q., Otting,G., Müller,M., Gehring,W. and Wüthrich,K. (1993) Determination of the Nuclear Magnetic Resonance Solution Structure of an Antennapedia Homeodomain-DNA Complex *J.Mol.Biol.*, **234**, 1084-1097.

8. Fraenkel,E. and Pabo,C.O. (1998) Comparison of X-ray and NMR structures for the Antennapedia homeodomain-DNA complex *Nat.Struct.Mol.Biol.*, **5**, 692-697.
